# Optimizing robotic cholecystectomy: improving cost-effectiveness with non-inferior safety and efficacy

**DOI:** 10.1007/s11701-026-03393-7

**Published:** 2026-07-20

**Authors:** Ron Dar, Zakhar Bramnick, Assi Drobot, Barak Levit, Wisam Sbeit, Moaad Farraj

**Affiliations:** 1 Department of General Surgery, Tzafon Medical Center, Lower Galilee, Israel; 2 Department of General Surgery, Galilee Medical Center, Nahariya, Israel; 3 Azrieli Faculty of Medicine, Bar Ilan University, Safed, Israel; 4 Department of Gastroenterology, Galilee Medical Center, Nahariya, Israel

**Keywords:** Robotic cholecystectomy, Multidimensional retraction, Robotic surgery

## Abstract

Achieving the critical view of safety during cholecystectomy is paramount for preventing bile duct injury but contingent on effective gallbladder retraction. Although robotic platforms offer enhanced dexterity and visualization, they present an opportunity to improve the static retraction methods common in laparoscopy. Herein, we describe our advanced dynamic multidimensional retraction (DMR) technique for three-port robotic cholecystectomy. This method uses the external joints of the robotic arms to provide continuous, multi vector exposure of the triangle of Calot, aiming to enhance surgical safety and efficiency. We conducted a retrospective review of the first 131 consecutive patients who underwent a three-port robotic cholecystectomy using our standardized DMR technique between January 2023 and July 2025. All procedures were performed on the DaVinci Xi or X platform by four surgeons during their initial robotic case series. Key demographic, intraoperative, and postoperative data were analyzed. Primary outcome measures included console times, length of hospital stay, intraoperative complications, and conversion rate to other surgical techniques. We performed 131 standardized robotic cholecystectomies via three-arm robot deployment by four teams. The mean patient age was 53 years; 50 were men and 81 were women. All teams started their robotic surgeries sequentially in a 4-months period in 2023. Most cases were elective, seven patients had acute cholecystitis, and 35 patients had a cholecystectomy integrated with other procedures like intraoperative cholangiogram, liver biopsy, repair of postoperative ventral hernia and one case of internal hernia repair after bariatric surgery. The mean console time was 57 min. There was no conversion to other surgical techniques. DMR is a safe and reproducible technique for three-port robotic cholecystectomy. By leveraging the robotic platform capabilities, our method provides non-inferior exposure of the critical anatomy. This advanced robotic approach represents a valuable tool for optimizing the surgical field and may improve safety and efficiency during robotic cholecystectomy. Further investigation in larger, comparative studies is warranted.

## Surgical highlights

Cholecystectomy operations require adequate gallbladder retraction.

The da Vinci robotic system incorporates instruments with several moving joints. These joints can be used to retract organs at two or more points.

## Abstract (key message)

**What is Conventional and what is Novel/Modified?**

• Robotic-assisted cholecystectomy is conventionally performed via four ports: one camera port and three instrument ports.

• A three-port robotic-assisted cholecystectomy (camera port and two instrument ports) was developed using unique robotic capabilities.

**What is the Implication, and What Should Change Now?**

• Three-port robotic-assisted cholecystectomy was shown to be safe and effective. This technique is teachable and reproducible, and may alter the way cholecystectomies are performed.

## Introduction

Robotics have gradually been implemented more often in the abdominal surgery field during the last two decades. However, the advantages of this method, such as precision, visibility and ergonomics, tend to be overshadowed by cost [1–5]. Cholecystectomy requires several well-described key actions, regardless of access. To carry out a safe and reproducible cholecystectomy, the surgical team must retract the fundus of the gall bladder upward toward the abdominal wall and create tissue tension before proceeding to dissection and cautery of the gall bladder neck. Since the introduction of laparoscopic cholecystectomy, several fundus retraction methods have been described in the literature.

In 2001, Endo et al. [6] described a new method of laparoscopic cholecystectomy using three trocars combined with suture retraction of the gallbladder. No major complications were noted, and 8% of the cases required a fourth trocar.

In 2012, an internal anchored retraction system was introduced in single-port laparoscopic cholecystectomy. None of the cases required the introduction of an additional trocar or conversion to conventional laparoscopy [7]. Uras et al. [8] published a paper in 2013 regarding an Endoloop gallbladder fundus retraction technique used in single-port laparoscopic cholecystectomy with promising results.

Parks et al. [9] described a transabdominal magnetic anchoring and guidance system in 2007. This system incorporates instruments, retractors, and a controllable intra-abdominal camera, all controlled magnetically through the abdominal wall. In vitro liver anchoring and camera operation proved successful, but later randomized comparison of different camera systems in natural orifice transluminal endoscopic operations showed no substantial effectiveness and a need for further system optimization for intra-abdominal surgery [10].

Robotic surgical systems were introduced and refined alongside these developments in laparoscopic retraction methods; to this day, robotics are used in many hospitals in Western countries. Fundus retraction is still a technical issue, and research on this subject remains ongoing. Chowbey et al. [11] published their first 100 robotic cases (using the Versus robotic surgical system) performed safely using three robotic arms with an additional subxiphoid laparoscopic assistant.

Fundus-first cholecystectomy has been described in the literature. Two recent works (Cengiz et al. [12], single center, 1,833 cases; Garzali et al. [13], meta-analysis) concluded that fundus-first laparoscopic cholecystectomy is safer and faster than conventional laparoscopic cholecystectomy.

The “performance envelope” is a term originating from aerodynamics that describes the theoretical structural capabilities of an aircraft when pushed to its designated maneuvering limits [14]. When extended to robotic surgical systems, this term references extracting the ultimate use of a system’s abilities up to its safety limits.

Our robotic surgery program, initiated in early 2023, initially mirrored the conventional laparoscopic cholecystectomy technique. This involved gallbladder retraction from the fundus to dissect the cystic artery and duct, aiming for the critical view of safety, as widely practiced. However, recognizing the unique capabilities of the robotic platform, we transitioned to an advanced technique designed to achieve what we term the “maximal view of safety”. This approach leverages the robot’s enhanced 3D visualization and multijointed flexibility of its instruments, allowing for a greater range of motion than the human wrist.

This refined technique employs the dynamic, multidimensional capabilities of the robotic arms to perform simultaneous retraction at two points and dissection with a third arm without an additional subxiphoid laparoscopic assistant. This dynamic robotic arm with multidimensional work allows for a more comprehensive and meticulous dissection of the gallbladder and surrounding structures.

Herein, we describe our technical approach to three-port robotic cholecystectomy, which we term “**Dynamic Multidimensional Retraction” (DMR)**. This technique leverages the external joints of the robotic arms, in addition to the intracorporeal wristed instruments, to provide continuous, adjustable, and multi-vector retraction of the gallbladder. This method facilitates simultaneous tissue manipulation and global retraction adjustments, creating an optimized and unobstructed surgical field for dissection. The objective of this approach is to maximize the exposure of Calot’s triangle, facilitate a definitive critical view of safety, and enhance operative efficiency and cost-effectiveness in a three-port setting.

## Methods

Following approval from the institutional review board, we conducted a retrospective review of a prospectively maintained surgical database. The study cohort included all 131 consecutive patients who underwent a three-port robotic cholecystectomy between February 2023 and July 2025. All procedures were performed by four senior surgeons who initiated their robotic surgery practice sequentially during a 4-month period in the study timeframe.

Data regarding demographics, mean age, sex, operation length, console time, integration with other operations, intraoperative or postoperative complications, previous abdominal surgery, and endoscopic retrograde cholangiopancreatography were collected.

All 131 operations were performed in a standardized manner, and specific equipment was used for three-arm robot deployment without an additional subxiphoid laparoscopic assistant. All operations used a Cadiere forceps for retraction and a robotic cautery hook for dissection. A robotic clip applier was used to ligate the cystic duct and artery. Suction was not regularly used.

### Step-by-step surgical technique

The procedure was performed under general anesthesia, with slight reversal of the Trendelenburg position and left tilt. All operations were carried out with the da Vinci Xi or X multiport system. A three-port technique was used with three 8 mm robotic ports placed in a diagonal line: the middle port in the midline about 20 cm from the subcostal margin of the mid-clavicular line and the other two in right-lower and left-upper abdomen about 8 cm apart.

First, retraction of the body of the gallbladder occurred upside, exposing the lateral part of its bed, then dissection was performed from the center of the gallbladder laterally to the fundus and the neck until good exposure and delineation were achieved. After releasing the lateral part, we divided the peritoneum from the medial border until complete dissection of the gall bladder wall, leaving it attached only at the cystic artery and cystic duct. After achieving a maximal view of safety, we clipped (two proximal clips and one distal, each) and divided the cystic artery and duct.

As shown in the media file, the two working arms served as multidimensional retractors using the arm joints; during gallbladder bed dissection, the joint of the Cadiere forceps was used to retract the gallbladder anteriorly to expose the dissection field, and during Calot’s triangle dissection, the cautery hook joint was used to retract the liver. Finally, the cystic duct and artery were ligated using a robotic medium-large clip applier. Gauze was used to absorb excess fluid, if needed.

## Results

### Patient demographics and case complexity

A total of 131 consecutive patients underwent three-port robotic cholecystectomy between February 2023 and July 2025. The cohort featured 81 (61.8%) women and 50 (38.2%) men, with a mean age of 53.2 ± 16.5 years (range: 19–87). Sixty-two patients (47%) presented with factors indicating increased surgical complexity: 35 patients (26.7%) had a history of previous major abdominal surgery or endoscopic retrograde cholangiopancreatography, 20 (15.3%) had undergone prior percutaneous cholecystostomy tube drainage, and 7 (5.3%) underwent surgery for acute cholecystitis. The more experienced first two teams exclusively performed these more complex cases.

## Intraoperative outcomes

All 131 procedures were successfully completed robotically with no conversions to laparoscopic or open surgery. The overall mean console time for the cohort was 57 min. An analysis by surgical team demonstrated a trend toward decreased operative time with increased case volume: Team A (*n* = 69 cases) had a mean console time of 47 min (range: 16–112), Team B (*n* = 40 cases) had a mean of 61 min (range: 18–140), and Teams C and D (*n* = 22 cases combined) had a mean of 65 min. Table 1

**Table 1:** Surgical operating teams

Team	Surgeries *n*	Acute *n* (%)	Drain *n* (%)	Mean console time	
				Mins	Range
A	69	5 (7%)	10 (14%)	47	16–112
B	40	2 (5%)	8 (20%)	61	18–140
C and D	22	0	0	65	

Concomitant procedures were performed with 16 patients (12.2%), including intraoperative cholangiography (*n* = 10), small incisional hernia repair (*n* = 2), liver biopsy (*n* = 3), and repair of an internal hernia (*n* = 1). Table 2

**Table 2:** Patients with past abdominal procedures and subdivision

Bariatric	Upper Gastrointestinal or Laparotomy	Umbilical Hernia	Endoscopic Retrograde Cholangiopancreatography	Gynecological
15	6	2	8	4

## Safety and postoperative outcomes

No major intraoperative complications occurred, defined as bile duct injury, visceral organ injury, or hemorrhage requiring blood transfusion. The 30-day postoperative period was uneventful, with no major complications (Clavien-Dindo Grade ≥ III). The median length of hospital stay was 1 day.

### Cost effectiveness

The costs were assessed using the manufacturer pricelists. Three-port robotic assisted cholecystectomy cost 30% less than four-port robotic assisted cholecystectomy (1483USD Vs. 1030USD accordingly). Table 3

**Table 3:** Operative cost (U.S. Dollars)

Equipment	Four-Port Robotic Assisted Cholecystectomy	Three-Port Robotic Assisted Cholecystectomy
Robotic Arm Drape	4 (390USD)	3 (293USD)
Trocar Cap	4 (140USD)	3 (105USD)
Monopolar Hook	205USD	205USD
Cadiere Grasper	2 (642USD)	1 (321USD)
Clip Applier	6 clips (106USD)	6 clips (106USD)
Total Cost	1483USD	1030USD

## Discussion

This multimedia article demonstrates our idea for a standardized three-port, fundus-first robotic cholecystectomy by a new technique, which we term DMR. Data were analyzed after 124 elective and seven acute operations performed by four teams in two peripheral hospitals that were trained sequentially in performing the operation using the same technique by the same proctor (the leader of the first team). Nearly half (47%) of the cases considered complex and performed by the first two teams, explaining the large diversity of console time among operations.

Given the constraints of limited robotic platform access to two sessions per week, a strategic approach to case allocation was adopted. To foster skill development across the surgical team, less complex cases were assigned to teams with less robotic experience. This approach provided valuable training ground for surgeons and support staff members to become familiar with the robotic system in a controlled setting.

Conversely, more complex cholecystectomy cases were preferentially assigned to our more experienced surgical teams. We contend that the robotic platform offers significant advantages in these challenging scenarios, rendering the procedures safer and more manageable. The enhanced 3D visualization, improved dexterity with wristed instruments, and greater surgical precision are particularly beneficial in cases with distorted anatomy or significant inflammation. These technological attributes facilitate meticulous dissection and may reduce the likelihood of complications such as bile duct injury. This strategy of case distribution ensures that patients with complex conditions receive the benefits of a more advanced surgical tool while allowing for the structured development of robotic surgical skills in the department.

This data suggests multidimensional retraction during three-port robotic cholecystectomy is safe and reproducible. The mean console time for the three-port technique was 57 min. No additional sub-xiphoid laparoscopic assistance was used, and no major complications occurred during the study period.

**Fig. 1:**
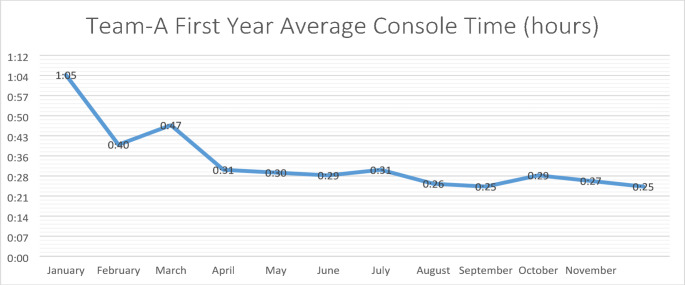
Example of Team-A surgeon average console time during the first year of robotic system implementation

This work has several limitations. Safety should be further validated in large scale studies, especially early in the learning curve. Our surgical teams cooperate regularly and share knowledge in order to enhance patient safety, and we recommend that surgeons adopting this technique will implement a supervised mentoring program. For several surgeons in the study operative teams, the robotic system implementation took place simultaneously with the introduction of multidimensional retraction with three-port robotic cholecystectomy. This may suggest that the learning curve data may not be accurate. For this reason Fig. 1 demonstrates a single surgeon’s learning curve as an example.

The primary endpoint of this study was reducing cost. We learned that operative costs can be substantially reduced when applying a three-port technique. This technique requires one fewer single use arm drape, one fewer single use trocar cannula seal, and one fewer grasper use (multiple use). As elaborated in the cost analysis, this translates to a 30% cost reduction per operation. This cost reduction may have a significant impact on any medical system worldwide that will implement a three-port robotic assisted cholecystectomy with the suggested advanced technique described here.

### Conclusion

Multidimensional retraction during three-port robotic cholecystectomy is cost-effective and reproducible. Using the robotic system and leveraging its performance envelope can become second nature for surgeons and should be considered worldwide. Our data shows non-inferiority regarding safety issues, and larger multicentric studies are warrented to confirm these findings.

## Data Availability

We have used case data after clearance from the institutional ethical board. data is saved in the author’s locked computer.

## Declarations

### Reporting Checklist

The authors have completed the SUPER reporting checklist.
